# User Experiences and Preferences Regarding an App for the Treatment of Urinary Incontinence in Adult Women: Qualitative Study

**DOI:** 10.2196/17114

**Published:** 2020-06-12

**Authors:** Nienke J Wessels, Lisa Hulshof, Anne M M Loohuis, Lisette van Gemert-Pijnen, Petra Jellema, Henk van der Worp, Marco H Blanker

**Affiliations:** 1 Department of General Practice and Elderly Care Medicine University of Groningen University Medical Center Groningen Groningen Netherlands; 2 Centre for eHealth and Wellbeing Research Department of Psychology, Health and Technology University of Twente Enschede Netherlands; 3 Faculty of Medical Sciences University of Groningen University Medical Center Groningen Groningen Netherlands

**Keywords:** ehealth, mobile applications, self-management, qualitative research

## Abstract

**Background:**

Although several apps are available to support the treatment of urinary incontinence (UI), little has been reported about the experiences and preferences of their users.

**Objective:**

The objective of this study was to explore the experiences and preferences of women using a mobile app for the treatment of UI and to identify potential improvements to the app. We developed this app for three types of UI: stress UI, urgency UI, and mixed UI.

**Methods:**

The participants in this qualitative study were women with self-reported stress UI, urgency UI, or mixed UI who used an app-based treatment to manage their condition for at least six weeks. Following the intervention, semistructured interviews were conducted to explore the participants’ experiences and preferences regarding the app. All interviews were audio-recorded, transcribed verbatim, and analyzed separately by two researchers.

**Results:**

Data saturation was reached after interviewing 9 women (aged 32-68 years) with stress UI (n=1, 11%), urgency UI (n=3, 33%), or mixed UI (n=5, 56%). Accessibility, awareness, usability, and adherence emerged as the main themes. On the one hand, participants appreciated that the app increased their accessibility to care, preserved their privacy, increased their awareness of therapeutic options, was easy to use and useful, and supported treatment adherence. On the other hand, some participants reported that they wanted more contact with a care provider, and others reported that using the app increased their awareness of symptoms.

**Conclusions:**

This qualitative study indicates that women appreciate app-based treatment for UI because it can lower barriers to treatment and increase both awareness and adherence to treatment. However, the app does not offer the ability of face-to-face contact and can lead to a greater focus on symptoms.

## Introduction

Approximately one-third of women with urinary incontinence (UI) seek medical attention [1]. In many cases, this low percentage can be explained by feelings of shame and embarrassment, perceptions regarding the normalcy of UI, and beliefs about the treatment options (or lack thereof) for UI. With increasing smartphone ownership, mobile health apps are providing a promising route to improving health care delivery and outcomes [2,3]. It is estimated that half of the approximately 3.4 billion people who use smartphones and tablets worldwide have downloaded a health app [4]. These apps play a particular role in reaching people who suffer from conditions that make them feel embarrassed or stigmatized, and they may help to lower barriers for women with UI [5]. Additionally, it has been shown that mobile app use can increase adherence to treatment advices, thereby improving outcomes and reducing health care costs [6,7]. Although several apps are available to support the treatment of UI, little has been reported about the experiences of their users, which is important for successful implementation of these apps [8]. We developed an app for the treatment of stress UI, urgency UI, and mixed UI. Recently, we have shown that this app is noninferior to care as usual, and usage of the app results in clinically relevant symptom improvement [9]. In the current study, we aimed to explore the experiences and preferences of women regarding the use of this app and to seek their opinions about potential areas for improvement.

## Methods

We chose a qualitative design for this study, as this design is especially suitable to explore and inquire into human experiences and preferences. Thematic analyses provide subthemes describing these experiences and preferences, as they are closely interwoven [10]. We conducted semistructured in-person interviews as part of the URinControl study, a mixed-methods study consisting of a randomized controlled trial (RCT) with extensive process evaluation to assess the expectations and experiences of patients and care providers regarding app-based treatment of UI in women [11]. The relevant medical ethics committee approved this study (M17.207954), and the COREQ guideline was followed in this report [12].

### Participants

Women were recruited through four primary care practices located in the northern part of the Netherlands. We selected practices that did not participate in the RCT to avoid the possibility of influencing the ongoing trial. We invited women who consulted their general practitioner (GP) for stress, urgency, or mixed UI in the past 10 years; were aged ≥18 years; self-reported UI at least twice a week; wanted treatment; and had access to a smartphone or tablet. The exclusion criteria were indwelling urinary catheter, urogenital malignancy, previous surgery for UI, treatment for UI in the previous year, terminal or serious illness, cognitive impairment or psychiatric illness (reported by their GP), overflow or continuous UI, pregnancy or recent childbirth (<6 months ago), or inability to complete a questionnaire in Dutch. The inclusion and exclusion criteria were identical to the criteria used in the RCT, enabling future triangulation (ie, combination) of the qualitative and quantitative data from the trial, to provide a complete picture of app usage. Purposive sampling was used to achieve diversity in age, UI type, educational level, and geographic location. Interviews were planned approximately 6 weeks after the participants received the login credentials for the app.

### The URinControl App

**Figure 1:**
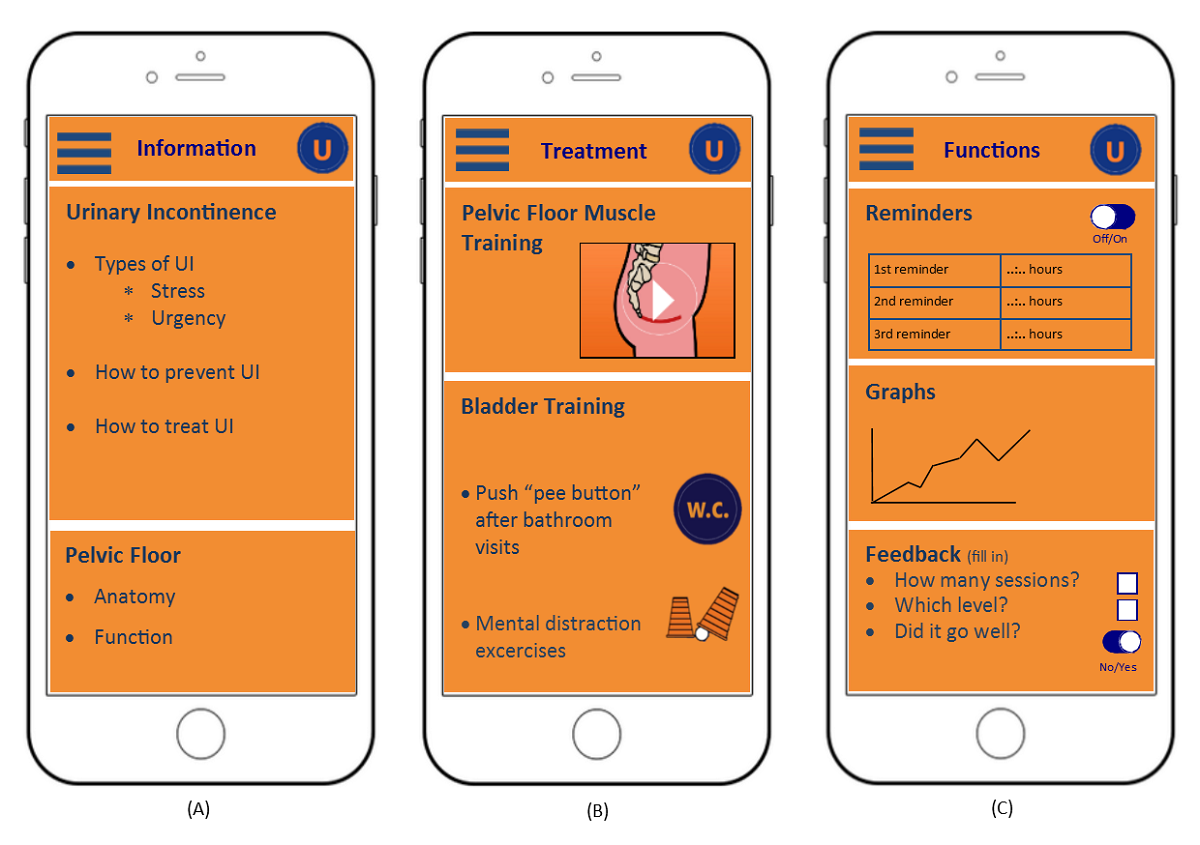
Representation of the contents of the URinControl app. (A) Information on both types of urinary incontinence, prevention and treatment options, as well as information on the anatomy and function of the pelvic floor. (B) Training programs for both stress and urgency urinary incontinence. (C) Functionalities of the app, including three reminder options, the graph function, and a patient feedback option. UI: urinary incontinence; W.C.: water closet.

The development of the URinControl app has been described elsewhere [11]. It provides step-by-step advice for treating stress UI, urgency UI, and mixed UI in a patient-friendly format based on the guidelines for treating female UI in primary care [13] (Figure 1).

### Basic Data Collection

After providing informed consent, each participant completed a short questionnaire to record their age, educational status, and duration of complaints. UI type was assessed according to the Three Incontinence Questions (3IQ), a 3-item questionnaire for classifying stress, urgency or mixed UI; mixed UI was further divided into stress-predominant UI, urgency-predominant UI, or mixed UI. To provide a description of the study population, we assessed UI severity with the International Consultation on Incontinence Modular Questionnaire Urinary Incontinence Short Form (ICIQ-UI-SF), a self-completed questionnaire (score 0-21; a higher score indicates greater severity). Furthermore, to measure self-perceived knowledge, comfort, and skill with finding, evaluating, and applying electronic health (eHealth) information to health problems, we used the eHealth Literacy Scale (eHEALS) [14], an 8-item questionnaire (score 8-40; a higher score represents greater literacy).

### Semi-Structured Interviews

To develop the semistructured interview guide, we used constructs of the Technology Acceptance Model (TAM) and the Mobile Application Rating Scale (MARS). The TAM is a widely used and validated model in both qualitative and quantitative studies of health apps. It was developed to explain and predict the acceptance and use of technology and is based on two explanatory constructs of users’ adoption: “perceived usefulness” and “perceived ease of use” [15]. The MARS is a validated and reliable scale that was developed to assess the quality of health apps based on four scales: engagement, functionality, aesthetics, and information quality [16].

We also gained insight into the actual app use, progress, and adherence of the participants by analyzing the automatic log data for the user interactions. These data were then used to personalize the interviews, enabling deeper discussion of themes (eg, if a participant had not used certain parts of the app). However, no attempts were made to combine the log data with the qualitative data. The interview guide (Multimedia Appendix 1) was tested in a pilot study before it was implemented.

The first author (NJW), a female physician trained to carry out qualitative interviews, conducted all the interviews. During each interview, the interviewer encouraged participants to talk freely about their experiences and provided opportunities to discuss subjects they felt had not been covered. The interviewer had no prior relationship with the participants. The second author (LH) was also present during the interviews and recorded additional notes (eg, on nonverbal communication).

The interviews took place at each participant’s GP practice, were recorded using a digital voice recorder, and were transcribed verbatim. Respondent validation was used to check the results. At regular points between interviews, we also conducted peer debriefings within the research group to evaluate the interviews and interview guide. Interviews were conducted until data saturation was reached, which was defined as the point at which no new codes were identified from 3 consecutive interviews.

### Analysis

Two researchers (NJW, LH) separately coded the transcripts using NVivo version 11 (QSR International). Thematic analysis was conducted according to the 6 phases of thematic analysis proposed by Braun and Clarke [17]. Data were analyzed by deductive and inductive approaches. The deductive coding framework was assembled using the TAM and the MARS [15] with inductive analysis reserved for responses that were unaligned to this model. After initital coding of each transcript, the researchers convened to compare codes and discuss emerging themes, by which they identified a set of four main themes that captured the essence of the interviews.

## Results

### Participants

Data saturation was reached after the ninth interview, after which no new participants were invited, and peer debriefing did not lead to changes in the interview guide. The 9 participants (aged 32 to 68 years) had suffered UI complaints for 18 to 312 months; 3 (33%) had urgency UI, 1 (11%) had stress UI, 2 (22%) had mixed UI, 2 (22%) had urgency-predominant mixed UI, and 1 (11%) had stress-predominant mixed UI. Of the 9 participants, 4 (44%) reported using incontinence pads daily. The median ICIQ-UI-SF score was 8 (range 2-13), indicating low to moderate self-perceived severity of UI. The eHealth literacy scores ranged from 17 to 33, indicating moderate to high self-perceived eHealth literacy (Table 1).

**Table 1:** Characteristics of the participants (N=9).

No	Age (years)	Educational level	BMI (k/m^2^)^a^	Type of UI^b^ (3IQ)^c^	Duration of UI (months)	Previous treatment for UI	Vaginal deliveries, n	ICIQ-UI-SF^d^ (total score)	eHEALS^e^ (total score)
1	49	Senior secondary vocational education	28.4	Stress-predominant mixed UI	180	None	1	12	32
2	64	Pre-university education	20.0	Urgency UI	60	Medication	2	7	32
3	68	Bachelor’s degree	19.5	Urgency-predominant mixed UI	36	PPT^f^	0	4	32
4	48	Senior secondary vocational education	36.9	Mixed UI	18	PPT	1	8	17
5	59	Master	32.7	Urgency UI	36	PPT	1	9	32
6	32	Secondary vocational education	22.2	Urgency-predominant mixed UI	60	PPT	2	7	31
7	37	Pre-university education	31.0	Urgency UI	72	PPT	0	13	31
8	53	Pre-university education	26.0	Stress UI	312	None	2	2	33
9	42	Bachelor’s degree	25.6	Mixed UI	120	PPT	1	9	30

^a^k/m^2^: kilograms per square meter.

^b^UI: urinary incontinence.

^c^3IQ: 3 Incontinence Questions.

^d^ICIQ-UI SF: International Consultation on Incontinence Modular Questionnaire Urinary Incontinence Short Form.

^e^eHEALS: eHealth Literacy Scale.

^f^PPT: pelvic physical therapy.

The automatically logged user data indicated that 28-59 days elapsed between app installation and the interview (Table 2), with actual app usage of 2-31 days (ie, the participant opened the app at least once on a day). The highest levels reached among the participants ranged from 0 to 6 out of a maximum of 15 levels.

**Table 2:** Overview of the log data.

Participant	Duration of usage (days)^a^	Days of usage^b^	Highest level reached (0-15)^c^	Use of reminders^d^
1	59	7	0	No
2	59	18	1	Yes
3	53	17	5	Yes
4	58	3	1	Yes
5	49	31	5	Yes
6	95	8	1	No
7	74	6	2	Yes
8	28	2	1	No
9	60	10	6	Yes

^a^Time in days between app installation and the interview.

^b^Days in which the app was opened at least one time.

^c^The highest level opened at least one time.

^d^Yes: turned on reminders at least once.

### Interviews

The interviews lasted 15-37 minutes, and thematic analysis of the transcripts resulted in four main themes concerning the experiences and preferences of women with the URinControl app (Table 3). These themes were accessibility, awareness, usability, and adherence.

**Table 3:** Participants' themes presented as main themes, subthemes, and meaningful codes.

Main themes and subthemes		Codes
**Accessibility**		
	Accessibility of care	Available 24 hours, 7 days a week
		Convenient
		Easily accessible
	Privacy	Keeps symptoms private
		Lowers the barrier to talk about UI
		Hinders privacy
**Awareness**		
	Awareness of symptoms	Confrontational
		Worsening of symptoms
		Coping strategies
	Awareness of therapy	Treatment options
		Effect of therapy
		Nothing new
**Usability**		
	Ease of use	Feeling familiar with app
		Clear
	Usefulness	Credible source
		Functionalities
		Graphs unclear
	Therapist support	App with therapist
		Information for health care provider
		App without therapist
**Adherence**		
	Challenges to adherence	Too busy
		Interference of daily activities
		Fit into daily routine
		Privacy
	Motivators for adherence	Improvement
		Progress in levels
		Reminders
		Motivational feedback

#### Theme 1: Accessibility

Accessibility concerns the women’s experience with access to UI health care with the app and the associated privacy. Most participants considered that the app was convenient and improved access to UI care and treatment. Information about the exercises was always available; therefore, the women could perform them when they wanted. Using the app was also considered less burdensome than receiving treatment from a health care provider:

You always have to make an appointment to see your physiotherapist. And everyone’s so busy these days that you think: why bother? And this gives you the support you need, because in theory it’s available 24 hours a day. And I really liked that.

Participant 7

Keeping UI symptoms private was important to some participants. They expressed that they found it difficult to discuss their symptoms with others and that they dreaded the internal examinations often performed by care providers. The accessibility of the app made it easier to receive treatment while keeping their complaints private:

I have control over the app, and I can keep it private. I find it difficult to talk about this with someone (and it definitely has to be a woman, not a man) and make it something so tangible. I want to keep it objective, deal with it in a simple, effective way and don’t make such a big thing about it.

Participant 2

Additionally, the app may have lowered barriers that prevented some women from talking about their symptoms with family and friends.

And it [incontinence] becomes less of a taboo topic [due to the app].

Participant 6

However, one participant with urgency UI stated that using the app made it difficult to keep her complaints secret from her spouse because he asked her why she used her phone so frequently.

#### Theme 2: Awareness

Participants reported gaining greater awareness of their symptoms and of potential coping and therapeutic strategies. Most participants had become used to their UI symptoms and had learned to live with them, and using the app increased their awareness. For some, this led to a realization that their symptoms were worse than they had previously thought:

[…] but I was also triggered by it. I thought: wow, this is really much worse than I thought, because I always adapt to this incontinence […]

Participant 2

Two participants with urgency UI experienced a worsening of symptoms after shifting their focus; however, others felt they became more aware of their dysfunctional coping strategies and how they could be addressed:

I just got even more incontinent; it became even more unpleasant.

Participant 5

I spoilt myself really by going to the toilet when I had to, and I had to learn not to.

Participant 2

Women became more aware of the conservative treatment options for UI, and in some instances, they changed their beliefs about the possible effects of these treatments. One woman with urgency UI who had received treatment for UI in the past was surprised by the impact of conservative treatment on her complaints:

If you persist and are consistent, there’s more you can do about it than I initially thought was possible. I was convinced that I would have to be operated on, but I find I’ve made a lot of progress, so I think if I carry on for a little longer, it can even get much better.

Participant 7

Several participants realized that to relieve their symptoms, they had to do the exercises for longer than they had initially hoped:

I have less difficulty now in making it to the bathroom, but I noticed that you have to train longer than you actually would want.

Participant 7

Unfortunately, the exercises only repeated previous training experiences for women who had already undergone pelvic floor muscle training; some of these participants had hoped that the app would offer new options.

#### Theme 3: Usability

Participants from various ages and educational levels stated that the app was easy to use, noting that it was self-explanatory and that they became familiar with it quickly. They appreciated its simplicity, clarity, and visual appeal:

The app was very clear, really easy to understand.

Participant 9

The women were positive overall about the usefulness of the app and stated they would recommend it to others. Concerning the different app functions, the information provided in the app was noted to be clear and useful. This was especially so for women who had not been treated for UI previously; in contrast, those who had already undergone pelvic physical therapy reported that it was repetitive. Some participants would have preferred less text with fewer difficult words. One participant appreciated that the app provided a credible source of information:

Of course, you can find films on YouTube, but you can never be sure whether they’re the right ones, because there’s a lot of rubbish on the internet. So that’s what I really like about it.

Participant 7

Participants appreciated the instructional videos in the app and found them more appealing and motivational than pictures. One participant reported that videos made it feel as if she was doing the exercises with someone else:

The videos are definitely useful. They show you how to exercise more efficiently. That makes it less a case of: [makes a sound expressing disgust] I have to exercise again. You just sit down and get on with it.

Participant 9

The distraction games included in the treatment program for women with urgency UI were used infrequently; most participants wanted greater variety or challenge to keep them engaged.

Concerning data presentation, some women found the included graph function to be unclear and stated that they did not understand how to interpret the graphs. Some merely glanced at the graphs, but others appreciated the statistics:

Yes, I think it’s interesting to look at it that way [the graphs]. […] You just can’t cheat.

Participant 3

There were also comments regarding input from health care providers. Several mentioned that the support and guidance of a pelvic physical therapist could add value, especially for older women who might have trouble navigating the app. One woman stated that face-to-face contact for ongoing training and support could improve her adherence. In other cases, participants suggested that information from the app could be used to inform their health care providers. A woman who had previously attended pelvic physical therapy reported that she missed the direct feedback, stating that the app would have been a valuable addition to that therapy:

I think it would be a good addition to a pelvic floor specialist, who does the exercises with you and lets you feel which muscles you have to tense. Because I really missed that.

Participant 5

Other women preferred to use the app alone because of poorly defined barriers to visiting pelvic physical therapists:

I feel there’s something holding me back from going to the physiotherapist specifically for that [the incontinence].

Participant 6

#### Theme 4: Adherence

Most participants experienced adherence and motivation issues; they found it difficult to perform exercises three times a day because they often felt they were too busy with daily activities. These women typically had trouble prioritizing treatment:

I’m always busy with the kids.

Participant 6

[About the app’s strengths] ‘Well, that it’s quick and easy to do. But not if you’ve got a busy job, or a very busy private life.”

Participant 8

The need for privacy also played a role when trying to fit the exercises into daily routines:

I actually wanted to do them [the exercises] quite frequently when I was on my own. So, when my children were in bed or after I’d taken them to school I thought: now, I’ve got all the time for it. So, I made it part of my own personal ritual.

Participant 7

Participants mentioned that noticing symptomatic improvement motivated them to persevere. One stated that being able to see progress in the exercise program motivated her, and another was motivated by the challenge to reach the highest level:

I still use it [the app] and I’m going to carry on with it. I’m a bit of an overachiever, so I want to get to the highest level.

Participant 9

Most of the participants stated that the inclusion of automated reminders was an important feature. However, although reminders were set by the participants, one woman commented that the timing was not always convenient:

At least for me, it [the reminder] acts as a trigger, like, oh yes, it’s that time again.

Participant 3

You get reminders to do these exercises, I liked that. And I made use of that option. But if you’re doing the washing up at that moment, or walking the dog, then you just click it away and an hour later you’ve forgotten about it.

Participant 4

Finally, one participant mentioned that she wanted feedback that was more stimulating and provided more praise to keep her motivated and engaged.

## Discussion

eHealth is an emerging area of health care technology with an ever-growing number of apps to assist and monitor patients with various health complaints. Research into the effectiveness and user experiences of these health apps is increasing. However, studies investigating apps for the treatment of UI are limited. We therefore aimed to explore the experiences and preferences of women regarding the use of an evidence-based mobile app that we had recently developed for the treatment of UI.

Literature on this topic is scarce. A review of the expectations and experiences of women concerning eHealth applications for UI [18] identified only one study of an online self-help program with email support of stress UI [19]. Recently, studies have been published on eHealth for women with stress UI, including their expectations of eHealth [20] and their experiences of an app focused on pelvic muscle training [21]. Notably, the two studies on experience with eHealth identified themes that overlapped considerably with those in our study [19,21].

### Principal Results and Comparison With Prior Work

Asklund et al [21] identified *enabling my independence* as a core theme reflecting desire to manage incontinence independently, with three subthemes of *something new!*, *keeping motivation up!*, and good *enough?* The *accessibility* and *something new!* themes are both related to accessibility of care, and the themes *adherence* and *keep motivation up!* both relate to treatment adherence. There is also overlap between the usability and *good enough?* themes; both involve uncertainty about performing exercises correctly without supervision. Björk et al [19] also identified a theme of *hidden but present*, which is closely related to our accessibility theme. However, neither of the earlier studies specifically identified an awareness theme, though Björk et al mentioned that participants had increased awareness of how to handle their incontinence. Given that the theme of increased symptom awareness was absent from earlier research and mainly arose from interviews of women with urgency UI, it may be a specific concern in this cohort.

The participants valued the easy access to the health care app throughout the day. Issues with privacy and talking about UI problems were raised by the participants, who indicated that they were attracted to the idea of receiving health care without consulting a health care provider. The app also made it easier for some women to talk about their complaints with friends or relatives. This is similar to the finding of Bjork et al [19], who reported that treatment for stress UI without face-to-face contact could break down barriers such as shame. However, some women still wanted to keep their symptoms private and preferred to use the app without consultation or care provider support. In contrast, other participants wanted greater care provider input from the start, particularly to confirm correct exercise performance and to promote adherence. These latter findings are consistent with studies in other fields indicating that participants prefer face-to-face contact in eHealth treatments [18,22].

Although other studies of UI apps have not reported greater self-awareness following app use, this outcome has been reported in studies of depression and chronic obstructive pulmonary disease [22,23]. Two of our participants expressed that greater self-awareness could be negative because they assessed the increased awareness as unpleasant and confrontational. The app treatment for urgency UI starts with monitoring toilet visits by pushing the “pee-button” and by advocating distraction techniques to extend the intervals between toilet visits. This treatment required these women had to change their behaviors and could induce feelings of insecurity and self-blame, especially if they were unsuccessful. Increased symptom awareness could therefore explain the worsening of symptoms. However, for other women, the app increased their awareness of the available treatment options and even changed some beliefs about the efficacy of conservative treatment. Several studies have shown that women are not always aware of treatment options or their effectiveness, which can adversely influence health-seeking behavior [1,24].

Perceived ease of use and perceived usefulness are the most important predictors of technology acceptance according to the TAM [15]. Women perceived our app to be both easy to use and clearly structured, and they appreciated that it was a credible information source, as shown previously [25]. However, while some women found the included information to be useful and easy to read, others wanted simpler text. This finding is consistent with research by Peng et al [26] and suggests the need for tailored information based on user preference.

Ensuring and monitoring treatment adherence is a major challenge when managing UI [27], and research has shown that adherence to eHealth apps is low in the absence of face-to-face contact [18]. Although the automatically logged user data showed low app use, this does not necessarily reflect true treatment adherence. Indeed, many women will undoubtedly perform the exercises without opening the app each time, especially as they become more practiced. Some participants would have liked more features to keep them engaged with the app, such as better feedback and a greater variety of distraction games. The importance of features supporting app engagement is stressed by studies showing that repeated use of health apps over time is low despite their wide availability [28,29]. The greatest barrier to adherence in this study was that the participants were too busy. Participants stated that they regularly forgot to perform the exercises and appreciated the reminder function, which was supported by the usage levels in the log data. Consistent with this finding, the importance of reminder functions in eHealth tools has been stressed in previous reports [23,25,26].

### Strengths and Limitations

We are not aware of any prior study of patient experience with mobile treatment for all three types of UI. To improve the robustness of our research, we integrated constructs of existing theoretical frameworks (TAM and MARS) in the interview guide, used log data to personalize the interviews, and confirmed the results by respondent validation. Moreover, we deliberately included a heterogeneous cohort based on age, level of education, and type of UI. However, women were recruited through only four practices in the northern part of the Netherlands. Therefore, the results may not be transferable to other GP populations. Moreover, although we analyzed the experiences of women with our app-based treatment over 6 weeks, we did not explore their experiences in the long term. Furthermore, although there were some indicators of differences in experiences between the UI subgroups (eg, increased symptom awareness led to worsening of symptoms for some women with urgency UI), the design of this study did not allow further analyses of the differences between these subgroups.

### Conclusions

In this qualitative study that incorporated log data, we showed that women tended to appreciate the URinControl app for treating UI. Use of this app may lower barriers to seeking treatment, increase self-awareness, and support treatment adherence. However, some women wanted more information about new therapies, more variety in the distraction games, contact with care providers, and improved feedback. Others wanted simplification of some areas, such as text with less detail and complexity and more understandable graphs. Notably, several women experienced a negative impact as awareness of their symptoms increased. These points of improvement will be taken into account with further development of the URinControl app. Furthermore, the experiences and recommendations outlined in this study can be used to optimize the implementation of this app in the future.

## Appendix

Multimedia Appendix 1 Interview guide.

## Abbreviations

3IQ: 3 Incontinence Questions
eHEALS: eHealth literacy scale
GP: general practitioner
ICIQ-UI SF: International Consultation on Incontinence Modular Questionnaire Urinary Incontinence Short Form
MARS: Mobile Application Rating Scale
PPT: pelvic physical therapy
RCT: randomized controlled trial
TAM: Technology Acceptance Model
UI: urinary incontinence
