# Supplementary material for: User Experiences and Preferences Regarding an App for the Treatment of Urinary Incontinence in Adult Women: Qualitative Study
Source: JMIR Mhealth Uhealth. 2020 Jun 12;8(6):e17114. doi: 10.2196/17114 (PMC7320303; doi:10.2196/17114)
Supplement: Multimedia Appendix 1 [file mhealth_v8i6e17114_app1.docx]

##### Appendix 1: Interview guide

| Main Question | Theory-subject |
| --- | --- |
| Expectations |  |
| Before you started, what were your thoughts on using an app for UI?   - *What were your expectations?* - *Which expectations were met/not met.* | TAM- perceived usefulness |
| Getting started |  |
| What were your experiences during the first time(s) that you used the app?   - *What was (un)clear?* - *How long did it take for you to feel familiar with the app?* - *Did you use the app on different devices?* - *Did you experience any technical problems with the app* | MARS- Functionality  TAM - Perceived Ease of Use |
| What are your thoughts on the appearance of the app?   - *Would you want to change anything?* - *Do you think the app is visually appealing/inviting? Why (not)?* - *Do you think the app is clearly structured? Why (not)?* - *Do you think the app is user-friendly? Why (not)?* | MARS- aesthetics  MARS- functionality  TAM - Perceived Ease of Use |
| App content |  |
| What are your ideas about the information provided by the app?   - *Regarding information on UI?* - *Regarding information on treatment option?* | MARS- information  TAM - Perceived Usefulness |
| How did you experience the:   - *Instructional video’s in the app?* - *Reminderfunction?* - *‘Pee-button”* - *Distraction games* - *Graphs* - *Did you explicitly not use any of the features? Why not?* | MARS- Functionality  MARS- Engagement  TAM - Perceived Usefulness |
| Looking back |  |
| What did you gain from using the app?   - *Did you learn something new?* - *Did you have any negative experiences?* - *What are strong points* - *What did you miss?* - *Are there changes in your experience of your symptoms?* | TAM - Perceived Usefulness |
| Would you recommend the app to other women with UI? Why (not)?   - *For which type of women do you think would the app be suitable or not suitable?* | TAM - Perceived Usefulness |
| Would you rather use an app or would you visit a pelvic floor therapist?   - *Why?* | TAM - Perceived Usefulness |
| Do you feel the app would fit in your daily life?   - *Why (not)?* - *What would make it easier to fit it into your daily life?* - *Do you think you will continue using the app? Why (not)?* | TAM - Perceived Usefulness |
| Improvement |  |
| Do you have any suggestions for improvement?   - *If you would change one thing about the app, what would it be?* | Suggestion(s) for improvement |
